# Pre-Existing Cardiovascular Conditions as Clinical Predictors of Myocarditis Reporting with Immune Checkpoint Inhibitors: A VigiBase Study

**DOI:** 10.3390/cancers12113480

**Published:** 2020-11-23

**Authors:** Roberta Noseda, Lorenzo Ruinelli, Linda C. van der Gaag, Alessandro Ceschi

**Affiliations:** 1 Division of Clinical Pharmacology and Toxicology, Institute of Pharmacological Sciences of Southern Switzerland, Ente Ospedaliero Cantonale, 6900 Lugano, Switzerland; Alessandro.Ceschi@eoc.ch; 2 Team Data Science & Research, Area ICT, Ente Ospedaliero Cantonale, 6500 Bellinzona, Switzerland; lorenzo.ruinelli@eoc.ch; 3 Dalle Molle Institute for Artificial Intelligence Research, 6928 Manno, Switzerland; linda.vandergaag@idsia.ch; 4 Faculty of Biomedical Sciences, University of Southern Switzerland, 6900 Lugano, Switzerland; 5 Department of Clinical Pharmacology and Toxicology, University Hospital Zurich, 8091 Zurich, Switzerland

**Keywords:** immune checkpoint inhibitor, myocarditis, cardiovascular conditions, (retrospective) matched case-control study, VigiBase

## Abstract

**Simple Summary:**

Up to 50% of myocarditis events developed in cancer patients upon treatment with immune checkpoint inhibitors (ICIs) are fatal. Therefore, identification of clinical risk factors predicting myocarditis onset during treatment with ICIs is important for the purpose of cardiac surveillance of high-risk patients. The aim of this retrospective matched case-control study was to assess whether pre-existing cardiovascular conditions were associated with the reporting of myocarditis with ICIs in VigiBase, the World Health Organization global database of suspected adverse drug reactions. Taking drugs labelled for the treatment of cardiovascular conditions as a proxy for concomitant cardiovascular risk factors and/or cardiovascular diseases, we found an association of moderate size between pre-existing cardiovascular conditions and the reporting of myocarditis with ICIs. Future prospective pharmacoepidemiological studies should assess the causal relationship between pre-existing cardiovascular conditions and myocarditis onset in a cohort of cancer patients followed during treatment with ICIs.

**Abstract:**

Although rare, immune checkpoint inhibitor (ICI)-related myocarditis can be life-threatening, even fatal. In view of increased ICI prescription, identification of clinical risk factors for ICI-related myocarditis is of primary importance. This study aimed to assess whether pre-existing cardiovascular (CV) patient conditions are associated with the reporting of ICI-related myocarditis in VigiBase, the WHO global database of suspected adverse drug reactions (ADRs). In a (retrospective) matched case-control study, 108 cases of ICI-related myocarditis and 108 controls of ICI-related ADRs other than myocarditis were selected from VigiBase. Drugs labeled as treatment for CV conditions (used as a proxy for concomitant CV risk factors and/or CV diseases) were found to be associated more strongly with the reporting of ICI-related myocarditis than with other ICI-related ADRs (McNemar’s chi-square test of marginal homogeneity: *p* = 0.026, Cramer’s coefficient of effect size: Φ = 0.214). No significant association was found between pre-existing diabetes and ICI-related myocarditis reporting (McNemar’s test of marginal homogeneity: *p* = 0.752). These findings offer an invitation for future prospective pharmacoepidemiological studies to assess the causal relationship between pre-existing CV conditions and myocarditis onset in a cohort of cancer patients followed during ICI treatment.

## 1. Introduction

Although rare, immune checkpoint inhibitor (ICI)-related myocarditis can be life threatening with a mortality rate up to 50% [1,2]. ICI-related myocarditis differs from classical acute myocarditis (which is generally caused by a post-viral immune response) in terms of both clinical presentations and outcomes [3,4]. Patients with ICI-related myocarditis are typically older than patients with non-ICI-related forms of myocarditis (64 years versus 34 years), have other chronic comorbidities in addition to their cancer, and show a higher mortality rate [5]. Identification of clinical risk factors predicting ICI-related myocarditis therefore is of primary importance, especially in view of increased ICI prescription [6].

To date, data on clinical risk factors for ICI-related myocarditis are limited and heterogeneous. From a multicenter registry, amongst 35 patients with ICI-related myocarditis, 34.3% had pre-existing diabetes and 42.9% had an angiotensin-converting enzyme inhibitor or an angiotensin receptor blocker as pre-ICI home cardiovascular (CV) medication [7]. Two distinct studies within the Food and Drug Administration Adverse Event Reporting System (FAERS) showed that the risk of ICI-related myocarditis is higher in female patients and in 75-years-old or older patients [8], and when either myositis, encephalitis or hepatitis develop concurrently with myocarditis upon ICI use [9]. In the Mayo Clinic database for cancer patients treated with ICIs, age over 80 years, a history of heart failure, and a history of acute coronary syndrome, were associated with a higher risk of ICI-related myocarditis, which occurred in 12 patients [10]. In a recent retrospective study of a cohort of 455 patients treated with ICIs for advanced melanoma from a single medical center, cross-referenced against a cohort of patients with similar characteristics extracted from VigiBase, the World Health Organization’s (WHO) pharmacovigilance database, ICI-related myocarditis occurred with a significantly older age distribution (median age: 69 years) than other ICI-related adverse drug reactions (ADRs) [11].

Our study aimed to assess whether pre-existing CV risk factors and/or CV diseases are associated with the reporting of ICI-related myocarditis in VigiBase. Using the description recorded in VigiBase of the role played by individual drugs in the occurrence of reported ADRs, concomitant drugs for CV conditions were taken as a proxy for the presence of pre-ICI CV risk factors and/or CV diseases. A (retrospective) matched case-control design (with the pre-existence of CV conditions as the independent variable and the reporting of ICI-related myocarditis as the dependent variable) was then adopted for comparing safety reports of ICI-related myocarditis, to control safety reports of ICI-related ADR(s) other than myocarditis from VigiBase.

## 2. Materials and Methods

### 2.1. Data Source

VigiBase (http://www.vigiaccess.org/) is the WHO global database of suspected ADRs, containing over 20 million safety reports submitted by physicians, pharmacists, healthcare professionals, patients/consumers, and pharmaceutical companies. To guarantee data quality, VigiBase employs a medical classification system (the WHO Medical Dictionary for Regulatory Activities, or MedDRA), by which information on suspected ADRs is organized in a uniform and structured hierarchical form. The system organ classes (SOCs) serve to group ADRs by their manifestation site or etiology, and preferred terms (PTs) are used to describe ADRs by single medical concepts. To support retrieval of MedDRA-coded information, VigiBase offers the Standardized MedDRA Queries (SMQs) tool, which encompasses prespecified sets of PTs. Drugs are identified either by their active ingredients through the WHO Drug Global dictionary [12], or by anatomical, therapeutic, and chemical codes (ATC).

Safety reports contain administrative features (such as database entry date, country of origin, reporter qualification, and safety report seriousness), patient characteristics (age and sex), ADR details (reported terms and outcomes), and drug descriptions (indication and role—suspected, concomitant, or interacting in relation to ADR onset) [13]. Safety report seriousness is taken as defined by the WHO-Uppsala Monitoring Centre and is based on the consequences the patient experienced from the suspected ADR(s). Serious safety reports, more specifically, are those that caused or prolonged hospitalization, led to death, were life threatening, disabled/incapacitated, or determined other clinically relevant conditions. Safety reports may mention multiple ADRs.

### 2.2. Data Retrieval and Selection

**Figure 1:**
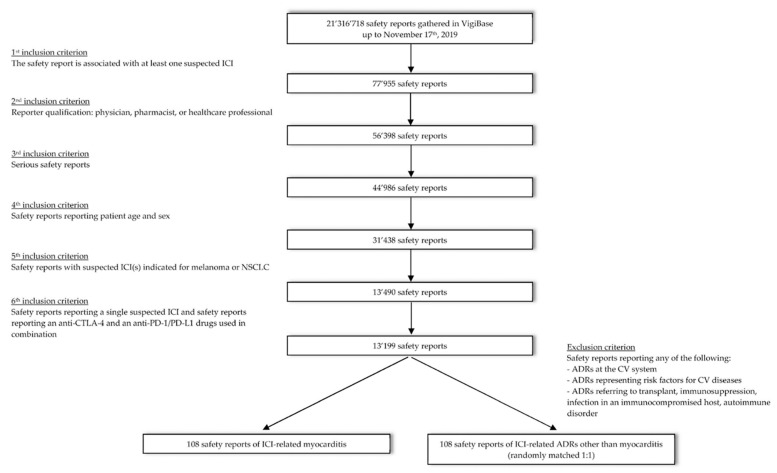
Consort diagram showing the multistep selection process applied to define the final dataset of safety reports eligible for the study.

Deduplicated safety reports gathered in VigiBase up to 17 November 2019, were retrieved, from which safety reports with ipilimumab, nivolumab, pembrolizumab, atezolizumab, avelumab, durvalumab, and cemiplimab (active ingredients) as suspected drugs, were selected. A multistep selection process was then used to define the group of safety reports eligible for the study (Figure 1). 

Safety reports entered by physicians, pharmacists, and healthcare professionals, and classified by reporters as serious were included; in view of the study design, safety reports missing information on patient sex and/or age were excluded. Safety reports with suspected ICI(s) pertaining to patients on ICI treatment because of melanoma or non-small cell lung cancer (NSCLC), the two main indications of ICIs, were selected in the next step (Table S1A). Subsequently, only safety reports with a single suspected ICI or an anticytotoxic T lymphocyte antigen-4 (CTLA-4, i.e., ipilimumab) and an antiprogrammed cell death-1/programmed cell death-ligand 1 (PD-1/PD-L1, i.e., nivolumab, pembrolizumab, atezolizumab, avelumab, durvalumab, or cemiplimab) used in combination, were retained for the study group. Safety reports with sequential treatments of two or more ICIs and safety reports with three suspected ICIs without information on dates of administration were excluded. We thereby assumed that a safety report indicating two cosuspected ICIs pertained to a patient who was on ICI combination treatment even if the safety report did not explicitly mention the starting dates for the individual ICIs as a confirmation.

Cases were defined as the safety reports of ICI-related myocarditis or autoimmune myocarditis (MedDRA PTs) from among the group of safety reports resulting from the selection process. Of the remaining group of non-cases, 1709 safety reports were excluded for indicating ICI-related ADR(s) at the CV system or ICI-related ADR(s) representing risk factors for myocarditis (and for CV toxicity in general), and/or for indicating an ICI-related transplant, immunosuppression, infection in an immunocompromised host, or autoimmune disorder (Figure 1 and Table S1B). The rationale behind the exclusion of these safety reports is that, on the one hand, reported ADRs at the CV system and risk factors for CV toxicity in general could be associated with the dependent variable of the present study (see Study Design below), while transplants, immunosuppression, and autoimmune disorders, on the other hand, are conditions that may affect the risk of ICI-related toxicity [14]. The remaining safety reports constituted the control group from which the controls for our study were sampled.

According to the Human Research Act (810.30, of 30 September 2011—status as of 1 January 2020), from the Federal Assembly of the Swiss Confederation, ethical approval was not required for use of safety reports retrieved from VigiBase (Art. 2: “It does not apply to research which involves anonymously collected or anonymized health-related data”).

### 2.3. Study Design

A (retrospective) matched case-control study was conducted, with cases as described above and controls taken from the overall control group. Patient sex, patient age, cancer type, and ICI treatment (i.e., monotherapy with an anti-CTLA-4 drug, monotherapy with an anti-PD-1/PD-L1 drug, or combination therapy with an anti-CTLA-4 and an anti-PD-1/PD-L1 drug), were used as matching criteria. The matching was done as random one-to-one matching, associating each case with a single control. The independent variable for the study was pre-existence of CV risk factors and/or CV diseases. Pre-existence of CV conditions was assessed using concomitant drugs of the anatomical, chemical, and therapeutic classes for the CV system (ATC C), for diabetes (ATC A10), and for obesity (ATC A08), as proxies. The dependent variable of the study was ICI-related myocarditis, that is, whether a safety report was a case or a control within the study.

Data processing for the study included data validation, a matching procedure, and descriptive analysis of the selected group of safety reports, and was implemented in Python (version 3.8.2). 

### 2.4. Statistical Analysis

From the safety reports in the case and control groups respectively, various demographic and clinical characteristics were summarized by counts and proportions. For establishing the relative risk (RR), per characteristic, for the case group compared to the control group, an odds-ratio (OR) approximation was employed to account for the size of the control group being imposed by the design of the study [15]. An RR larger than 1.00 indicates that the characteristic is more likely to be found in the case group than in the control group; an RR smaller than 1.00 indicates that the characteristic is more likely in the control group. To establish a 95% confidence interval (CI) for the (approximate) RR per characteristic, a normal approximation of the natural logarithm of the observed OR was used. For analyzing the similarity of the rankings of the categories of the multicategorical characteristic of organ toxicity in the case and control groups respectively, the Mann–Kendall test of rank correlation was used at a significance level of α = 0.05 [16].

For analyzing the association between ICI-related myocarditis and pre-existing CV risk factors and/or CV diseases in the study group of safety reports, McNemar’s chi-square test of marginal homogeneity was employed. This non-parametric test for paired categorical data was applied to a 2 × 2 table tabulating all case-control pairs under study, and focused on the discordant pairs for testing whether the two marginal probabilities per category of the independent variable are the same; by disregarding the concordant pairs, the test was less affected by the matched design of the study than the standard chi-square test. Under the null hypothesis of marginal homogeneity, McNemar’s test statistic has a chi-squared distribution with a single degree of freedom. For evaluating the null hypothesis, a (two-tailed) significance level of α = 0.10 was used to allow for a reasonable power of the study in view of a small study-group size. Upon rejection of the null hypothesis, Cramer’s Φ coefficient of effect size was employed as a measure of strength for the association found between the independent and dependent variables.

All statistical tests were performed using the public web-based tool GraphPad (https://www.graphpad.com/quickcalcs/).

## 3. Results

The selection of safety reports from VigiBase as described above resulted in 110 safety reports indicating ICI-related myocarditis. Two of these safety reports were removed because no matching controls were found among the selected safety reports. After matching therefore, a study group resulted with 108 cases of ICI-related myocarditis and 108 controls of ICI-related ADRs other than myocarditis.

### 3.1. Safety Report Demographic and Clinical Characteristics

Table 1 shows the demographic and clinical characteristics of the safety reports included in the study. In the group of 216 safety reports in all, the four matching variables had the following characteristics: the median patient age was 68 years (interquartile range, IQR: 60–74 years); as to patient sex, 62.0% of the safety reports involved males; 59.3% of the patients had melanoma for their cancer type, and 69.4% received anti-PD-1/PD-L1 monotherapy for their ICI regimen. The case group compared to the control group showed a higher approximate RR of drug-related fatalities (seriousness criterion: “Led to death”, RR 2.61, 95% CI: 1.37–4.96), of life-threatening ADRs (seriousness criterion: “Was life-threatening”, RR 6.65, 95% CI: 2.21–20.0), and of fatalities and life-threatening ADRs combined (RR 4.71, 95% CI: 2.58–8.60). Regarding the country of origin, cases compared to controls were more frequent from the United States (RR 2.01, 95% CI: 1.12–3.61) and less frequent from Japan (RR 0.57, 95% CI: 0.32–1.02). In both groups of cases and controls respectively, ICIs were the sole suspected drugs in 98 (90.7%) safety reports.

**Figure 2:**
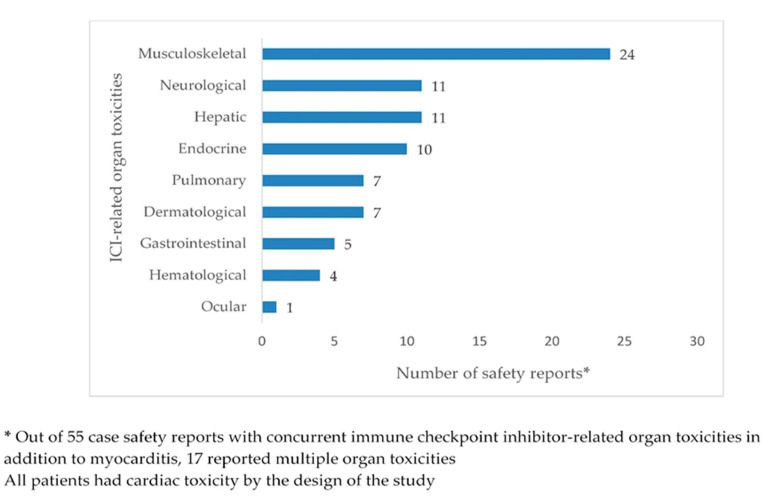
Immune checkpoint inhibitor-related organ toxicities reported in addition to myocarditis in the group of case safety reports.

**Figure 3:**
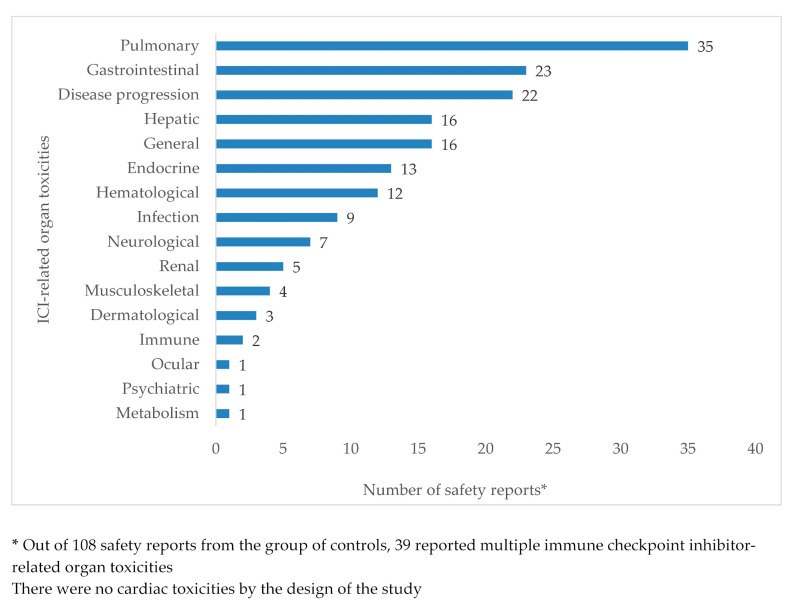
Organ toxicities reported with immune checkpoint inhibitors in the group of control safety reports.

Figure 2 and Figure 3 show, per organ, the numbers of toxicities reported as ICI related in the case and control groups, respectively. As, by the design of the study, all safety reports in the case group and none of the safety reports in the control group indicated myocarditis as an ICI-related ADR, counts of cardiac toxicity were not represented in the two figures. From the case group, 53 (49.1%) safety reports indicated myocarditis as the only ICI-related ADR; the remaining 55 (50.9%) case safety reports mentioned a total of 80 ICI-related organ toxicities in addition to myocarditis (Figure 2). In the group of control safety reports (Figure 3), a total of 170 ICI-related organ toxicities were indicated; 69 (63.9%) controls reported single organ toxicity, while 39 (36.1%) reported multiple organ toxicities. Pulmonary, gastrointestinal, hepatic, endocrine, and hematological toxicities were reported in more than 10% of control safety reports each. Disease progression was reported in 22 (20.4%) controls, and general toxicities (including pyrexia, fatigue, and asthenia, among others) in 16 (14.8%) controls. Upon Mann–Kendall testing of rank correlation at the significance level of α = 0.05, the null hypothesis of independent rankings was not rejected (*p* = 0.34).

### 3.2. Statistical Analysis of Association

For the 108 case-control pairs in the study, Table 2A tabulates the four possible combinations of categories of the independent and dependent variables, with pre-existence of CV risk factors and/or CV diseases (ATC class C) as the independent variable. The numbers on the main diagonal of the table report the numbers of case-control pairs with concordant categories for the pre-existence of CV conditions; the numbers on the secondary diagonal report the numbers of pairs with discordant categories. The number of discordant pairs, in which exactly one of a matched pair of case and control had a pre-existing CV condition, was 29. McNemar testing at the significance level α = 0.10 led to rejection of the null hypothesis of marginal homogeneity (*p* = 0.026). Using Cramer’s Φ coefficient of effect size, the observed deviation from marginal homogeneity was found to be an association of moderate size (Φ = 0.214) between the independent and dependent variables [17].

Table 2B similarly tabulates the possible combinations of categories of the independent and dependent variables, now with the pre-existence of diabetes (ATC class A10) as the independent variable, and shows the associated counts over the 108 case-control pairs. The number of discordant pairs was 10. McNemar testing at the significance level of α = 0.10, did not lead to rejection of the null hypothesis of marginal homogeneity (*p* = 0.752). The overall study group of cases and controls did not include any safety report mentioning the use of antiobesity preparations (ATC class A08) and therefore did not allow an analysis of their potential association with the independent variable.

In view of the 108 case-control pairs of our study, McNemar testing at the preset significance level of ɑ = 0.10 had a statistical power of 1 − β = 0.70. At a significance level of α = 0.15, McNemar testing would have had a statistical power of 1 − β = 0.80.

## 4. Discussion

Our matched case-control study of safety reports selected from VigiBase as described above, revealed an association of moderate size between pre-existing CV conditions and the reporting of ICI-related myocarditis. Drugs labeled as treatment for CV conditions, used as a proxy for concomitant CV risk factors and/or CV diseases, were associated more strongly with the reporting of ICI-related myocarditis than with the reporting of any other ICI-related ADR in VigiBase. No significant association was found between pre-existing diabetes and ICI-related myocarditis reporting. As antiobesity preparations were not mentioned in any of the included safety reports, our study did not allow an analysis of a potential association of their use with the reporting of ICI-related myocarditis. The latter two findings may be due to the relatively small size of the group of safety reports used in the study.

Observational studies to date, of real-world clinical data from patient registries, pharmacovigilance databases, and claim databases, have yielded heterogeneous information on clinical risk factors for ICI-related myocarditis [7,8,9,10,11,18]. In a retrospective cohort study within the Mayo Clinic database of cancer patients treated with ICIs, patient age over 80 years was found to be associated with an increased risk of ICI-related myocarditis, which occurred in a total of twelve patients [10]. Patient age of 75 years or older emerged as a clinical risk factor for ICI-related myocarditis in a pharmacovigilance study within FAERS. In this study, a broader-scoped definition of myocarditis (captured by the MedDRA high-level term non-infectious myocarditis, which includes, in addition to myocarditis and autoimmune myocarditis, other forms of myocarditis, such as hypersensitivity myocarditis, lupus myocarditis, and radiation myocarditis) was employed than in our study. In the multiple logistic regression models used, moreover, age, sex, and ICI use were used as covariates, while other potential confounders, such as cancer type and the use of concomitant drugs, were not [8]. An older age distribution (median: 69 years) was observed among patients with ICI-related myocarditis in a study integrating data from electronic medical records from a single centre with those from VigiBase [11]. In this study, in addition to patient age, also patient sex was assessed as a potential risk factor for ICI-related myocarditis (and other ICI-related ADRs in general), yet no significant associations were found. Female sex was associated with a significantly higher risk of ICI-related myocarditis in the aforementioned pharmacovigilance study in FAERS [8].

Combination treatment is a well-established clinical risk factor for ICI-related myocarditis [7,8]. From an investigation of cancer type as a potential risk factor, in a United States claim database of 252 patients treated with ICIs and presenting with myocarditis, renal and lung cancers were found to be associated with ICI-related myocarditis onset. In the same study, also diabetes mellitus and liver diseases as underlying comorbidities were found to be risk factors for ICI-related myocarditis [18]. Diabetes (as a CV risk factor) and angiotensin-converting enzyme inhibitors or angiotensin receptor blockers (as pre-ICI home drugs for underlying CV diseases) were more commonly found in patients with ICI-related myocarditis than in patients without such an ADR in a case-control study within the medical records from a multicenter registry [7]. Data from the Mayo Clinic further revealed that a history of heart failure and a history of acute coronary syndrome were also associated with a higher risk of ICI-related myocarditis [10].

With respect to ICI toxicities more in general, age subgroup analyses in randomized clinical trials (RCTs) suggested comparable safety profiles of ICIs regardless of patient age [19]. From observational studies, however, information on ICI toxicity in older patients is more controversial, sometimes suggesting that older patients may be at higher risk of ICI toxicity [20]. A recent retrospective study at the Mayo Clinic institution on 476 cancer patients with either melanoma or NSCLC receiving anti-PD-1 therapy showed that women are more likely to develop ICI-related toxicity of any grade than men [21], consistent with increased immunoglobulins production mediated by female sex hormones and higher incidence of autoimmune diseases in women [22]. Different toxicity profiles with anti-PD-1 ICIs have been observed in different cancer types in a systematic revision of RCTs, with gastrointestinal and dermatological ICI-related ADRs occurring with higher frequency in melanoma patients, and pneumonitis developing more frequently in patients with NSCLC [23]. Lastly, ICIs used in a combination regimen are found to be associated with a worse safety profile than ICIs in monotherapy, with a higher frequency of adverse events [24]. The multistep selection process used in our study was designed to mitigate, as much as possible, the inherent heterogeneity of safety reports collected in VigiBase, which originates from reporters with different qualifications and from countries with different pharmacovigilance regulations [13]. Through our selection process, we defined a study group composed of ICI-related safety reports entered by physicians, pharmacists, and healthcare professionals (responsible in 2017 for increased reporting in VigiBase of ICI-related myocarditis [25]) and classified as serious, where the latter criterion was used to minimize the reporting bias of serious ADRs due to mandatory reporting obligations by regulatory authorities in countries such as the United States of America [26]. The matched case-control design further allowed us to adjust for possible biases from the selection process and from age, sex, ICI regimen, and cancer type as potential confounding factors of ICI-related toxicity.

In our study, we observed that the safety reports of ICI-related myocarditis involved mostly males, likely reflecting the fact that, in RCTs, a higher proportion of men receive ICI treatment than women [27]. The median age of patients who were reported with ICI-related myocarditis was 68 years, in line with a higher incidence of melanoma and NSCLC in older adults [28,29]. Anti-PD-1/PD-L1 ICIs, which are approved for a wide spectrum of indications [6], were most frequently reported as suspected of having caused myocarditis. The countries of origin mentioned in the safety reports in our study were quite heterogeneous, with most of cases and controls originating from the United States of America (likely because of earlier approval and earlier usage of ICIs), followed by Japan and the European Union. We found a statistically significant difference from the distributions of countries of origin between cases and controls. From the United States of America, the study group included considerably more case safety reports of ICI-related myocarditis than control safety reports of ICI-related ADRs other than myocarditis; in contrast, from Japan, case safety reports were less numerous than controls. These findings suggest that American citizens, compared to for example citizens of Japan, may be exposed to an increased risk of ICI-related myocarditis. This increase in risk is likely due to health conditions, lifestyle, and family history, which are established risk factors for heart disease in the United States of America [30], in addition to the patient characteristics controlled by study design. In a previous study of patients undergoing anti-PD-1 immunotherapy for metastatic melanoma, immune-related adverse events were shown to correlate with improved survival [31]. While ICI-related adverse effects are mostly reversible and often do not require specialized management [32], ICI-related myocarditis is known to be life-threatening and sometimes fatal [1]. Our study of the selected group of safety reports from VigiBase confirmed the higher seriousness of ICI-related myocarditis compared to other ICI-related ADRs, with cases more frequently resulting in fatal outcomes or life-threatening conditions than controls.

While in VigiBase information on pre-existing and/or concomitant comorbidities is not systematically collected, concomitant drugs and the indications for which they are used are recorded (albeit with the variable accuracy inherent to a voluntary reporting system) [13]. Information on concomitant drugs has been used before as a proxy for patient comorbidities in pharmacovigilance studies within VigiBase [33]. Recent research published by the WHO-Uppsala Monitoring Centre in fact supports our definition of the independent variable using concomitant drugs to infer a patient’s underlying diseases. The researchers considered concomitant diseases among the patient risk factors for ADRs reported in VigiBase, defined subgroups of such diseases using concomitant drug indications as proxies, and proposed subgroup disproportionality analysis as a statistical methodology for risk characterization in the ensuing patient subpopulations for ADR onset [34].

In the majority of safety reports in our study group, ICIs were the sole drugs indicated as suspected of causing either myocarditis or other ADR onset. To reduce a potential competition bias from other antineoplastic drugs playing a role in ADR onset, a sensitivity analysis was performed of the association found between pre-existing CV conditions and the reporting of ICI-related myocarditis. For this purpose, safety reports mentioning antineoplastic drugs other than ICIs (captured by ATC code L01) were excluded from the study group. Since patients with underlying autoimmunity may be at higher risk of ICI-related ADRs [14], we also excluded safety reports mentioning immunostimulants (ATC L03), immunosuppressors (ATC L04), and/or corticosteroids for systemic use (ATC H02). Safety reports with corticosteroids for systemic use as concomitant drugs to treat underlying autoimmune diseases or because of a transplant were excluded, while safety reports with corticosteroids for systemic use for the management of ICI-related toxicity were retained. To the reduced study group of 89 case-control pairs, McNemar's chi-square test of marginal heterogeneity was applied at a significance level of α= 0.10, as before. The null hypothesis was again rejected (*p* = 0.046) and the association between the independent variable (pre-existing CV conditions defined as concomitant drugs for the CV system— ATC C) and the dependent variable was once more found to be of moderate size (Cramer’s coefficient: Φ = 0.212; Table S2). The consistency of effect size in both the original and the reduced study groups suggests that the association found between pre-ICI drugs for the CV system and the reporting of an ICI-related myocarditis was little affected by other pre-existing conditions for which drugs of the ATC codes L01, L03, L04, or H02 are indicated.

In accordance with previous studies, which reported about half of ICI-related myocarditis cases presenting with also other organ toxicities (most frequently myositis and myasthenia gravis [7,35]), we found in our study that 50.9% of myocarditis cases were co-reported with additional ICI-related ADRs; the co-reported ADRs were mainly musculoskeletal and neurologic toxicities, which were also found in a previous pharmacovigilance study in FAERS [9]. In our study group, controls were found to present with a somewhat different pattern of organ toxicities, albeit without statistical significance, with pulmonary, gastrointestinal, hepatic, endocrine, and hematological toxicities reported in more than 10% of the selected control safety reports.

The study reported in this paper has several strengths, giving support to our conclusions. As it has a wide geographical coverage, VigiBase allowed us to involve different trends in spontaneous reporting of ICI-related myocarditis across countries. By using the MedDRA PTs of myocarditis and autoimmune myocarditis to define the dependent variable moreover, we increased study specificity [36]. The choice of McNemar’s chi-square test of marginal homogeneity (instead of, for example, the more standard chi-square test of independence) allowed us to use a well-matched control group of limited size to handle the relative rarity of ICI-related myocarditis [15]. Despite the relatively small number of cases, McNemar testing was associated with a reasonable balance of significance and statistical power.

Yet, our findings need to be interpreted in the context of some limitations. VigiBase is the global largest source of post-marketing safety reports of ADRs. Being dependent on spontaneous reporting however, safety reports from different countries and different types of reporters increase the heterogeneity of the recorded information, in terms of timeliness, completeness, and quality [13]. In addition, VigiBase is known to suffer from reporting biases, confounding issues, and scant clinical richness, including risk factors [13]. We decided to control these issues by carefully restricting our study group and by using a matched study design. Selecting only safety reports of melanoma and NSCLC patients (the two main ICI indications) and including only serious safety reports (which are the most represented in VigiBase on ICIs [37]), as controlling measures, may however have affected the generalizability of our findings. As VigiBase further lacks information on the total number of patients treated with a specific drug and provides information only on the number of patients treated with a certain drug and being reported with one or more ADR(s), the incidence of a drug/ADR pair of interest cannot be estimated from VigiBase. The retrospective case-control design of our study and the statistical tests of association used to assess reporting differences of myocarditis versus other ICI-related ADRs were tailored to the VigiBase context, and could not provide conclusions as to the causal nature of the association found between the independent and dependent variables. Lastly, instead of querying VigiBase to generate hypotheses, as has been widely done in pharmacovigilance studies on ICIs through disproportionality analysis [38], we used VigiBase to test a hypothesis. Due to the limitations inherent to the data source mentioned above, our findings do not as yet allow drawing definitive conclusions but should rather be interpreted as supporting a hypothesis that deserves further investigation.

## 5. Conclusions

In line with previous studies reporting a higher risk of ICI-related myocarditis with pre-ICI home angiotensin-converting enzyme inhibitor or angiotensin receptor blocker treatment, a history of heart failure and/or a history of acute coronary syndrome [7,10], the results of our study provide further support to the hypothesis that pre-existing CV risk factors and/or CV diseases are associated with the reporting of myocarditis in patients treated with ICIs. In clinical practice, cancer patients with pre-existing CV conditions could thus be exposed to higher risk of developing myocarditis upon treatment with ICIs, and might benefit from a more intensive cardiac surveillance. Our findings offer an invitation for future prospective pharmacoepidemiological studies to assess the causal relationship between pre-existing CV conditions and myocarditis onset in a cohort of cancer patients followed during ICI treatment.

## Figures and Tables

**Table 1:** Demographic and clinical characteristics of the study group of safety reports.

Characteristics	Cases (*n* = 108)	Controls (*n* = 108)
*Patient sex* *		
Female	41 (38.0)	41 (38.0)
Male	67 (62.0)	67 (62.0)
*Patient age* *		
<65 years	42 (38.9)	42 (38.9)
≥65 years	66 (61.1)	66 (61.1)
Median (IQR),	68 (60–74)	68 (60–74)
Range (in years)	7–83	7–83
ICI regimen*		
Anti-CTLA-4 monotherapy	5 (4.6)	5 (4.6)
ipilimumab	5	5
Anti-PD-1/PD-L1 monotherapy	75 (69.4)	75 (69.4)
nivolumab	48	48
pembrolizumab	19	19
atezolizumab	7	7
durvalumab	1	1
Combination of anti-CTLA-4 and anti-PD-1/PD-L1	28 (25.9)	28 (25.9)
ipilimumab and nivolumab	25	25
ipilimumab and pembrolizumab	3	3
*Cancer type* *		
Melanoma	64 (59.3)	64 (59.3)
Non-small cell lung cancer	44 (40.7)	44 (40.7)
*Reporting year*		
2013	-	1 (0.9)
2014	-	1 (0.9)
2015	1 (0.9)	8 (7.4)
2016	8 (7.4)	18 (16.7)
2017	26 (24.1)	26 (24.1)
2018	38 (35.2)	25 (23.1)
2019	35 (32.4)	29 (26.9)
*Country of origin*		
United States of America	42 (38.9)	26 (24.1)
Japan	28 (25.9)	41 (38.0)
European Union	26 (24.1)	27 (25.0)
Australia	4 (3.7)	1 (0.9)
United Kingdom	3 (2.8)	6 (5.6)
Switzerland	2 (1.9)	-
Canada	2 (1.9)	6 (5.6)
Turkey	1 (0.9)	-
Montenegro	-	1 (0.9)
*Type of reporter*		
Physician	68 (63.0)	74 (68.5)
Healthcare professional	34 (31.5)	30 (27.8)
Pharmacist	6 (5.6)	4 (3.7)
*Seriousness criteria*		
Caused or prolonged hospitalization	35 (32.4)	53 (49.1)
Led to death	37 (34.3)	18 (16.7)
Was life-threatening	22 (20.4)	4 (3.7)
Disabled/incapacitated	-	1 (0.9)
Determined other clinically relevant conditions	14 (13.0)	32 (29.6)
*Co-suspected drugs (in addition to ICIs)*		
Not reported	98 (90.7)	98 (90.7)
Reported	10 (9.3)	10 (9.3)
Median number per safety report (IQR)	1 (1–1.7)	1 (1–2.7)
ATC L01 Antineoplastic agents	6 (5.6)	4 (3.7)
*Concomitant drugs*		
Not reported	59 (54.6)	67 (62.0)
Reported	49 (45.4)	41 (38.0)
Median number per safety report (IQR)	4 (2–8)	5 (3–9)
ATC C^1^ Cardiovascular system	30 (27.8)	17 (15.7)
C01 Cardiac therapy	6 (5.6)	3 (2.8)
C02 Antihypertensives	3 (2.8)	-
C03 Diuretics	10 (9.3)	4 (3.7)
C04 Peripheral vasodilators	1 (0.9)	1 (0.9)
C07 Beta blocking agents	8 (7.4)	7 (6.5)
C08 Calcium channel blockers	8 (7.4)	2 (1.9)
C09 Agents acting on the renin-angiotensin system	12 (11.1)	6 (5.6)
C10 Lipid modifying agents	11 (10.2)	12 (11.1)
ATC A10 Drugs used in diabetes	5 (4.6)	7 (6.5)
ATC A08 Antiobesity preparations	-	-
ATC C and A10	5 (4.6)	6 (5.6)

Data are count (%); * Matching variable; ^1^ In both groups of cases and controls, some safety reports indicated multiple concomitant drugs from ATC class C. Abbreviations: IQR, interquartile range; ICI, immune checkpoint inhibitor; CTLA-4, cytotoxic T-lymphocyte antigen-4; PD-1, programmed cell death-1; PD-L1, programmed cell death-ligand 1; ATC, anatomical, therapeutic, chemical.

**Table 2:** Counts of the case-control pairs with respect to pre-existence of cardiovascular risk factors and/or cardiovascular diseases.

**(A).**				
		**CASES**		
		With concomitant drugs of ATC C	Without concomitant drugs of ATC C	*Totals*
CONTROLS	With concomitant drugs of ATC C	9	8	*17*
	Without concomitant drugs of ATC C	21	70	*91*
	*Totals*	*30*	*78*	*108*
**(B).**				
		**CASES**		
		With concomitant drugs of ATC A10	Without concomitant drugs of ATC A10	*Totals*
CONTROLS	With concomitant drugs of ATC A10	1	6	*7*
	Without concomitant drugs of ATC A10	4	97	*101*
	*Totals*	*5*	*103*	*108*

Abbreviations: ATC, anatomical, therapeutic, chemical; C, cardiovascular system; A10 drugs used in diabetes.

## Supplementary Materials

The following are available online at https://www.mdpi.com/2072-6694/12/11/3480/s1, Table S1: Search criteria for safety-report selection in VigiBase, Table S2: Counts of the case-control pairs of the restricted study group, with respect to the pre-existence of cardiovascular conditions.
