# Supplementary material for: Pre-Existing Cardiovascular Conditions as Clinical Predictors of Myocarditis Reporting with Immune Checkpoint Inhibitors: A VigiBase Study
Source: Cancers (Basel). 2020 Nov 23;12(11):3480. doi: 10.3390/cancers12113480 (PMC7700285; doi:10.3390/cancers12113480)
Supplement: Supplementary file 1 [file cancers-12-03480-s001.pdf]

cancers

Supplementary materials: Pre-Existing Cardiovascular Conditions as Clinical Predictors of Myocarditis Reporting with Immune Checkpoint Inhibitors: A VigiBase Study

Roberta Noseda, Lorenzo Ruinelli, Linda C. van der Gaag and Alessandro Ceschi

Table S1. Search criteria for safety report selection in VigiBase.

**A) ICI indication**
Melanoma → including the following (as coded according to the MedDRA classification system):<br>Melanoma<br>Malignant melanoma<br>Metastatic malignant melanoma<br>Metastatic melanoma<br>NSCLC → including the following (as coded according to the MedDRA classification system):<br>NSCLC<br>NSCLC recurrent<br>NSCLC metastatic
**B) ICI-related ADRs that led to safety report exclusion from the control group**
• ICI-related ADR(s) at the CV system included in the MedDRA SOCs "cardiac disorders" and "vascular disorders", and/or<br>• ICI-related ADR(s) representing risk factors for myocarditis (and for CV toxicity in general), listed in the MedDRA SMQs "hyperglycaemia/new onset diabetes mellitus", "ischaemic heart disease", "dyslipidaemia", "hypertension", "chronic kidney disease", or reported as (MedDRA PTs) "obesity" and "tobacco user", and/or<br>• ICI-related "transplant", "immunosuppression", "infection in an immunocompromised host", or "autoimmune disorder" (MedDRA PTs)

Abbreviations: ICI, immune checkpoint inhibitor; MedDRA, Medical Dictionary for Regulatory Activities; NSCLC, non-small cell lung cancer; CV, cardiovascular; SOC, system organ class; SMQ, standardized MedDRA query.

Table S2. Counts of the case-control pairs on the restricted study group with respect to the pre-existence of cardiovascular risk factors and/or cardiovascular diseases in.

		**CASES**		
		With concomitant drugs of ATC C	Without concomitant drugs of ATC C	*Totals*
CONTROLS	With concomitant drugs of ATC C	2	7	*9*
	Without concomitant drugs of ATC C	18	62	*80*
	Totals	*20*	*69*	*89*

Abbreviations: ATC, anatomical, therapeutic, chemical; C, cardiovascular system.
